# Testing the joint effects of arbuscular mycorrhizal fungi and ants on insect herbivory on potato plants

**DOI:** 10.1007/s00425-024-04492-1

**Published:** 2024-07-30

**Authors:** Xoaquín Moreira, Lucía Martín-Cacheda, Gabriela Quiroga, Beatriz Lago-Núñez, Gregory Röder, Luis Abdala-Roberts

**Affiliations:** 1 Misión Biológica de Galicia (MBG-CSIC), Apartado de Correos 28, 36080 Pontevedra, Galicia Spain; 2 Centro de Investigaciones Agrarias de Mabegondo (CIAM), Apartado de Correos 10, 15080 A Coruña, Spain; 3 Institute of Biology, University of Neuchâtel, Rue Emile-Argand 11, 2000 Neuchâtel, Switzerland; 4 Departamento de Ecología Tropical, Campus de Ciencias Biológicas y Agropecuarias, Universidad Autónoma de Yucatán, Apartado Postal 4-116, Itzimná, 97000 Mérida, Yucatán México

**Keywords:** Ants, Arbuscular mycorrhizal fungi, Phenolic compounds, Plant defenses, *Solanum tuberosum*, Volatile organic compounds

## Abstract

**Main conclusion:**

Ants, but not mycorrhizae, significantly affected insect leaf-chewing herbivory on potato plants. However, there was no evidence of mutualistic interactive effects on herbivory.

**Abstract:**

Plants associate with both aboveground and belowground mutualists, two prominent examples being ants and arbuscular mycorrhizal fungi (AMF), respectively. While both of these mutualisms have been extensively studied, joint manipulations testing their independent and interactive (non-additive) effects on plants are rare. To address this gap, we conducted a joint test of ant and AMF effects on herbivory by leaf-chewing insects attacking potato (*Solanum tuberosum*) plants, and further measured plant traits likely mediating mutualist effects on herbivory. In a field experiment, we factorially manipulated the presence of AMF (two levels: control and mycorrhization) and ants (two levels: exclusion and presence) and quantified the concentration of leaf phenolic compounds acting as direct defenses, as well as plant volatile organic compound (VOC) emissions potentially mediating direct (e.g., herbivore repellents) or indirect (e.g., ant attractants) defense. Moreover, we measured ant abundance and performed a dual-choice greenhouse experiment testing for effects of VOC blends (mimicking those emitted by control vs. AMF-inoculated plants) on ant attraction as a mechanism for indirect defense. Ant presence significantly reduced herbivory whereas mycorrhization had no detectable influence on herbivory and mutualist effects operated independently. Plant trait measurements indicated that mycorrhization had no effect on leaf phenolics but significantly increased VOC emissions. However, mycorrhization did not affect ant abundance and there was no evidence of AMF effects on herbivory operating via ant-mediated defense. Consistently, the dual-choice assay showed no effect of AMF-induced volatile blends on ant attraction. Together, these results suggest that herbivory on potato plants responds mainly to top-down (ant-mediated) rather than bottom-up (AMF-mediated) control, an asymmetry in effects which could have precluded mutualist non-additive effects on herbivory. Further research on this, as well as other plant systems, is needed to examine the ecological contexts under which mutualist interactive effects are more or less likely to emerge and their impacts on plant fitness and associated communities.

**Supplementary Information:**

The online version contains supplementary material available at 10.1007/s00425-024-04492-1.

## Introduction

Mutualisms are ubiquitous drivers of ecological processes and species evolution (Thompson 2006; Bascompte and Jordano 2007; Bascompte 2019). One of the key attributes of these interactions is their large degree of variation in specialization (i.e., obligate to highly generalist) and, relatedly, the presence of asymmetry in mutualist partner effects (Chamberlain et al. 2014; reviewed by Bronstein 2015). Much of the early work focused on highly specialized mutualisms in a pairwise fashion (Bronstein et al. 2003; Kiers et al. 2011), but subsequent research started embracing multi-species generalist interactions characterized by strong lability and context-dependency, both now well-recognized hallmarks of mutualisms (Chamberlain et al. 2014; Yule et al. 2020). Plant-associated mutualistic interactions comprise most of the known or described mutualisms and exhibit these features (Bascompte and Jordano 2014; Weber and Agrawal 2014; Chomicki et al. 2019; Doré et al. 2023). Plants can interact to varying extents with multiple mutualist partners (e.g., one or more functional groups or guilds) belonging to the same type of mutualism as well as with different types of mutualists (reviewed by Bronstein 2015). Studies addressing the latter case are nonetheless rare despite the documented occurrence and presumed importance of diversity in co-occurring plant-associated mutualistic interactions. Filling this research gap is crucial to determine how different types of plant mutualists jointly shape ecological and evolutionary processes.

Plant associations with different types of mutualists often implicate above- and belowground interactions (Pineda et al. 2010, 2013; De Deyn 2017). Within the aboveground realm, ant–plant mutualisms are one of the most common and conspicuous (e.g., over 19,000 plant species rely on ants as defenders and seed dispersal vectors; Luo et al. 2023). This interaction offers significant benefits to both parties. Ants aggressively attack herbivores that try to feed on the plants and clean plant surfaces to reduce the chances of pathogens taking hold (Heil and Mckey 2003; Rosumek et al. 2009; reviewed by Anjos et al. 2022). In return for these defensive services, plants often provide ants with food (e.g., nectar or extrafloral nectaries, and food bodies) and shelter (e.g., hollow thorns or domatia) (Rico-Gray and Oliveira 2007; Weber and Keeler 2013).

Arbuscular mycorrhizal fungi (AMF), one the other hand, form symbiotic relationships with plants and dominate the belowground realm of plant-associated mutualisms (documented on approximately 80% of known plant species; Bardgett and van der Putten 2014; Wipf et al. 2019). They improve plant nutrient and water uptake, which generally leads to enhanced plant growth and higher biomass production (e.g., Rausch et al. 2001; Courty et al. 2015; Begum et al. 2019), and, in return, plants supply AMF with carbohydrates derived from photosynthesis, which represent their primary energy source as they are obligate biotrophs (reviewed by Wipf et al. 2019). Importantly, AMF can also benefit plants via indirect effects, e.g., by negatively affecting antagonists. For example, they can trigger the upregulation of defense-related genes and the production of defensive compounds like phytohormones ( Song et al. 2011; Pedone-Bonfim et al. 2013; Vannette et al. 2013; Frew et al. 2018), which can deter herbivores or reduce their ability to feed effectively (reviewed by Pozo and Azcón-Aguilar 2007; Rasmann et al. 2017; Frew et al. 2022). Further, they can also alter other plant traits mediating interactions with the third trophic level, such as plant-based rewards or cues providing information on prey availability to parasitoids and predators (Bronstein 2015; Keller et al. 2018), including ants.

To date, the effects of AMF on ant–plant interactions have received very little attention and the plant traits mediating interaction outcomes are usually overlooked. One of the few exceptions is a study by Keller et al. (2018), who found that rhizobia increased ant abundance via increased production of extrafloral nectaries in *Chamaecrista fasciculata*, therefore, providing evidence of mutualist interactive effects. Linkages between AMF and aboveground tri-trophic interactions may also arise AMF-induced changes in plant traits conveying information to carnivores. In particular, AMF have been shown to drive changes in plant volatile organic compound (VOC) emissions (reviewed by Rasmann et al. 2017), and in doing affect parasitoid and predator abundance or behavior (Heil 2011; Papantoniou et al. 2022). That said, studies on plant VOC effects on ants are less common and have usually involved more specialized systems in which plants offer rewards or shelter (reviewed by Nelson et al. 2019), making it difficult to tease apart the effects of VOCs from these other traits (but see Rasmann et al. 2014; Schettino et al. 2017). The effects of AMF on ant–plant VOC-mediated interactions could therefore represent an important missing piece in our understanding of linkages between below- and aboveground plant mutualisms.

*Solanum tuberosum* L. (Solanaceae) is an herbaceous annual plant that is currently one of the most important crops globally (FAOSTAT 2023). In southwestern Europe, potato plants in agricultural settings host a diverse community of both specialist and generalist insect herbivores, including mainly leaf chewers such as the Colorado potato beetle *Leptinotarsa decemlineata*, the beet armyworm *Spodoptera exigua*, and *Agriotes* spp. (Radcliffe et al. 2007; Alyokhin et al. 2013). Previous work has reported on the defensive role against insect herbivores of secondary metabolites such as phenolic compounds (Friedman 2006; Kumar et al. 2016), and VOCs acting as induced resistance in neighboring potato plants (Vázquez-González et al. 2022, 2023; Martín-Cacheda et al. 2023). Potato plants are colonized by several species of arbuscular mycorrhizal fungi (AMF), including *Rhizophagus irregularis*, a common AMF in potato plantations in southwestern Europe (Buysens et al. 2017). This fungus can increase potato resistance to leaf-chewing herbivores such as *Trichoplusia ni* (Schoenherr et al. 2019). In addition, *R. irregularis* establishes generalist interactions with ant species such as *Lasius niger* and *Formica rufa*, which have been reported to defend potato plants against herbivores (Schettino et al. 2017). To date, however, the mechanisms by which these mutualists influence herbivory (e.g., mycorrhizae effects via effects on direct defenses vs. ant-mediated indirect defense) and the occurrence of non-additive dynamics have not been studied. In particular, the role of herbivore-induced VOCs as cues for ant attraction appears to be important based on recent work on this species (Schettino et al. 2017), but the influence of AMF on these dynamics remains unstudied.

In this study, we investigated the independent and interactive effects of AMF and ants on insect leaf herbivory and associated plant traits in potato (*Solanum tuberosum*) plants. We conducted a field experiment where we factorially manipulated the presence of AMF (two levels: control and mycorrhization) and ants (two levels: exclusion and presence). To tease apart plant trait-mediated mechanisms, we tested for AMF effects on plant phenolic compounds, which serve as direct defenses, as well as on VOCs which could act as direct (e.g., herbivore repellents) and/or indirect (e.g., ant attractants) defenses. We further investigated AMF VOC-mediated effects on ants by conducting a greenhouse choice experiment testing for VOC effects on ant attraction using volatile blends mimicking those emitted by control and AMF-inoculated plants in the field. In doing so, we aimed to answer the following: (1) Do AMF and ants influence insect herbivory, and are such effects independent or interactive (i.e., non-additive)? (2) Are these above- and belowground mutualist effects explained by changes in plant traits involved in direct (phenolic compounds) and/or indirect (ant attraction via VOCs) defense? We hypothesized that the effects of AMF on herbivory would primarily act through increased ant recruitment in response to AMF-induced changes in VOC emissions. This would lead to non-additive dynamics, where AMF effects are disproportionately stronger or only occur in the presence of ants, and conversely, ant effects are disproportionally stronger in the presence of AMF. Overall, this study unique insight into plant-associated mutualists by teasing apart the independent and joint effects of different types of mutualists on herbivory, and further exposing plant traits responsible for these below- and aboveground interaction linkages.

## Materials and methods

### Greenhouse test of mycorrhizal treatment effects on plant traits

#### Experimental design and measurements

In June 2022, we sowed 144 tubers of three potato varieties (*S. tuberosum* L. cultivar Agria, Baraka, Desiree, i.e., 48 plants of each variety) in 4-L pots previously disinfected with NaClO and containing soil with a 1:1 sand/peat mixture previously autoclaved for three consecutive days using a double autoclaving bag. At the time of sowing, to half of the pots, we added 5 g/pot of a commercial inoculum of *R. irregularis* (“mycorrhization” hereafter) provided by Atens biotech company (Tarragona, Spain). For the other half of the plants, we added 10 mL of a filtrate of the leftover inoculum (20–30 g) to provide the general microbial population that the inoculum contains but without the AMF propagules (“control” hereafter). Plants were grown in a glasshouse under controlled light (minimum 10 h per day, photosynthetically active radiation = 725 ± 19 μmol m^−2^ s^−1^) and temperature (10 °C at night, 25 °C in the day), and watered three times a week. In August 2022, four weeks after applying the mycorrhizal treatment, we recorded stem height and number of leaves for all plants, and collected constitutive (i.e., basal levels) aboveground VOCs from 24 plants of each mycorrhizal treatment (48 plants in total) following Rasmann et al. (2011). In addition, we collected three fully expanded leaves and oven-dried them at 40 ºC for 48 h to quantify constitutive phenolic compounds.

### VOC collection

We bagged plants with a 2 L Nalophan bag, and trapped VOCs on a charcoal filter (SKC sorbent tube filled with Anasorb CSC coconut-shell charcoal) for 2 h using a Sidekick 224-52MTX pump (0.25 L min^−1^ airflow of technical air N_2_O_2_). We eluted traps with 150 μL dichloromethane (CAS#75–09-2; Merck, Dietikon, Switzerland) to which we had previously added one internal standard (naphthalene CAS#91–20-3, 200 ng in 10 μL dichloromethane). We then injected 1.5 μL of the extract for each sample into an Agilent 7890B gas chromatograph (GC) coupled with a 5977B mass selective detector fitted with a 30 m × 0.25 mm × 0.25 μm film thickness HP-5MS fused silica column (Agilent, Santa Clara, CA, USA). We operated the injection into the GC in pulsed splitless mode (250 ºC, injection pressure 15 psi) with helium as the carrier gas. The GC oven temperature program was: 3.5 min hold at 40 ºC, 5 ºC min^−1^ ramp to 230 ºC, and then a 3 min hold at 250 ºC post run (constant helium flow rate 0.9 mL min^−1^). The transfer line was set at 280 ºC. In the MS detector (EI mode), a 33–350 (m/z) mass scan range was used with MS source and quadrupole set at 230 ºC and 150 ºC, respectively. We identified volatiles using either commercial pure standards or mass spectrum comparisons with NIST library. Kováts indices, calculated relative to the retention times of a series of n-alkanes (C_8_-C_20_, Sigma-Aldrich, Merck KGaA, Darmstadt, Germany) analyzed under the same chromatographic conditions, were compared with those reported in the literature for supporting the identification step. We measured total emission of individual VOCs using normalized peak areas and expressed it as nanograms per hour (ng h^−1^). We obtained the normalized peak area of each individual compound by dividing their integrated peak areas by the integrated peak area of the internal standard (Moreira et al. 2021; Abdala-Roberts et al. 2022), in order to standardize for variation in the sample volume during the elution process. Reported values for individual VOCs should thus be considered as naphthalene-equivalent nanograms of compound released by each plant per hour (Rasmann et al. 2011). The total emission of VOCs of each sample was then obtained by summing the concentrations of individual VOCs.

### Quantification of phenolic compounds

We ground leaves collected in the greenhouse using liquid nitrogen to obtain a single pooled sample. We then extracted phenolic compounds from 20 mg of dry pulverized leaf tissue with 1 mL of 70% methanol in an ultrasonic bath for 15 min, followed by centrifugation (Moreira et al. 2014). We then transferred the extracts to chromatographic vials and performed ultra-high-performance liquid chromatography analyses. For phenolic quantification, we used ultra-high-performance liquid-chromatograph (UHPLC Nexera LC-30AD; Shimadzu Corporation, Kyoto, Japan) equipped with a Nexera SIL-30AC injector and one SPD-M20A UV/VIS photodiode array detector. The compound separation was carried out on a Kinetex™ 2.6 µm C18 82–102 Å, LC Column 100 × 4.6 mm, protected with a C18 guard cartridge. The flow rate was 0.4 mL min^−1^ and the oven temperature was set at 25 ºC. The mobile phase consisted of two solvents: water-formic acid (0.05%) (A) and acetonitrile-formic acid (0.05%) (B), starting with 5% B and using a gradient to obtain 30% B at 4 min, 60% B at 10 min, 80% B at 13 min and 100% B at 15 min. The injection volume was 5 µL. For phenolic compound identification, we used an ultra-performance liquid chromatography coupled with electrospray ionization quadrupole (Thermo Dionex Ultimate 3000 LC) time-of-flight mass spectrometry (UPLC-Q-TOF–MS/MS) (Bruker Compact™, Billerica, MA, USA). We identified two groups of phenolic compounds: flavonoids and caffeic acids. We achieved the quantification of these phenolic compounds by creating calibration curves using known concentrations of standard compounds (0.16, 0.8, 4, 20, 100 and 500 μg mL^−1^). These curves were then used to determine the concentration of the analyte in the sample by comparing its response (e.g., peak area) to that of the standards. We used rutin as the standard for flavonoids and caffeic acid as the standard for caffeic acids (Moreira et al. 2018a). We expressed phenolic compound concentrations in mg g^−1^ tissue on a dry weight basis.

### Statistical analyses

We ran linear models to assess the impact of mycorrhizal treatment (two levels: control and mycorrhization), plant variety (three levels: Agria, Baraka, Desiree), and their interaction (all fixed factors) on plant growth (height, number of leaves), direct defenses (using separate models for each group of phenolic compounds), and indirect defenses (total VOCs, potentially influencing ant abundance). In all cases, we used data from plants prior to ant exclusion. We also included plant height as a covariate in the preliminary models for phenolics and VOCs to account for size differences that may influence compound concentrations. However, it was never found to be significant and was subsequently removed. All models were run with PROC GLM in SAS ver. 9.4 (SAS Institute, Cary, NC, USA) (Littell et al. 2006). For VOCs, we additionally ran a permutational multivariate analysis of variance (PERMANOVA) model testing for the effects of mycorrhizal treatment, plant variety, and their interaction on the composition of volatile emissions which could also affect ant recruitment. For this, we used compound abundances and analyses were based on 10,000 permutations performed with the ‘vegan’ package in R ver. 4.0.2 software (Oksanen et al. 2016). To visualize PERMANOVA results, we conducted a principal coordinates analysis based on Bray–Curtis pairwise dissimilarities and graphed the centroids of each mycorrhizal treatment effect (Moreira et al. 2021). We also identified VOCs that correlated strongly (R^2^ > 0.50) with the first two ordination axes (using ‘envfit’ in vegan; Oksanen et al. 2016) and displayed these relationships using biplot arrows with length scaled to R^2^ values.

### Field test of mycorrhization and ant exclusion effects on ant abundance and herbivory

#### Experimental design

**Fig. 1:**
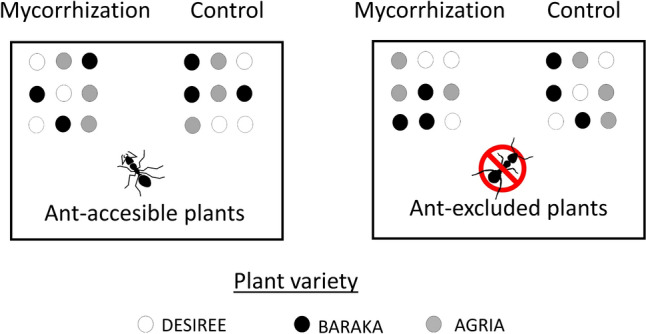
Schematic representation of one block in the experimental design, showing the ant exclusion treatment (two levels: exclusion and presence) as the whole factor; mycorrhizal treatment (two levels: control and mycorrhization) as the split factor; and three plant genetic entries (i.e., varieties) as the split–split factor. Each experimental unit (the combination of mycorrhizal and ant exclusion treatments) consisted of nine plants in three parallel rows of three plants each (three of each potato variety)

The day after leaf and VOC sampling was concluded, we moved all potted plants to a nearby field site with presence of ant mounds (42.41º N, 8.64º W, Pontevedra, Spain) following a randomized split–split design replicated in four blocks, with an ant exclusion treatment (two levels: exclusion and presence) as the whole factor, mycorrhizal treatment (two levels: control and mycorrhization) as the split factor, and plant variety as a split–split factor (Fig. 1). The purpose of blocking was to account for spatial variability in unmeasured factors, therefore, increasing power of our test of main effects. Each experimental unit (i.e., combination of mycorrhizal and ant exclusion treatments) consisted of nine plants in three parallel rows of three plants each (three of each potato variety; Fig. 1). Within each unit, we separated neighboring plants by approximately 20 cm, and experimental units were spaced at least 1 m apart, with the positioning of plants within the unit being randomized. All blocks were separated by at least 3 m. In total, we used 144 potato plants, corresponding to four blocks × two ant exclusion treatments × two mycorrhizal treatments × three plant varieties × three replicates of each variety. The ant exclusion treatment involved a weekly application of sticky paste (Tanglefoot®; Tanglefoot Company, Grand Rapids, MI, USA) around the upper surface of the pots of assigned plants. This sticky paste is the most common method to exclude ants from plants (e.g., Mooney 2007; Moreira et al. 2012) and is made with all-natural ingredients (gum, resins, vegetable oil, and wax).

### Ant abundance and herbivory measurements

From August to October 2022, we carried out 1-min ant observations of each plant for a total of 10 surveys throughout this period (approximately every week) (mean ± SE per survey: 0.279 ± 0.015 ants plant^−1^, range: 0.208–0.347 ants plant^−1^). All observations were conducted from 09:00 to 12:00 AM, which is when ant activity is highest. We used the sum of the number of ants across surveys per plant for statistical analyses. In late October 2022, at the end of the growing season, we randomly collected 10 fully expanded leaves of roughly the same age (based on position along branch, color, and consistency) for each plant to assess herbivory. Since plants produce several leaf flushes but also retain leaves from previous flushes, our herbivory estimates likely represented a combination of late-season herbivory and cumulative herbivory over the growing season, i.e., our sampling included different leaf cohorts. Most of the damage observed on the collected leaves were due to chewing insects (> 90% of sampled leaves). We photographed all leaves with a Samsung Galaxy A30s (25 effective megapixels, 4 × digital zoom) and estimated the percentage of leaf area consumed by chewing insects (“leaf-chewing herbivory” hereafter) using BioLeaf—Foliar Analysis™ (Brandoli Machado et al. 2016). We used the average value across leaves per plant for statistical analyses. Insect leaf damage averaged less than 5% (Fig. S1), consistent with typical rates observed in potato plants in our study area (X. Moreira, personal observation). Still, these levels have been shown to impose significant detrimental effects in potato plants such as reductions in tuber and biomass production (Moreira et al., unpublished data). Immediately after leaf sampling for herbivory, we also collected roots from 10 plants of each mycorrhizal treatment to estimate the percentage of root length colonized by AMF. To differentiate AMF structures, we stained the roots with Trypan blue following the procedure described by Phillips and Hayman (1970). We then used the gridline intersect method in those roots to determine the extent of mycorrhizal colonization (Giovannetti and Moose 1980). Unfortunately, we cannot test for genetic variation in mycorrhizae colonization because the plants selected for quantification were randomly chosen and all came from only two of the three varieties (Baraka and Agria).

### Statistical analyses

We tested for effects of mycorrhizal treatment (two levels: control and mycorrhization), ant exclusion treatment (two levels: exclusion and presence), plant variety (three levels: Agria, Baraka, Desiree), and their two- and three-way interactions (all fixed factors) on ant abundance and leaf-chewing herbivory (percent leaf area consumed) using a linear mixed model in PROC MIXED solving for a split–split design (ant exclusion: whole-plot factor; mycorrhizal treatment: split-plot factor). We also included the block as a random factor in these models. We additionally included the ant exclusion by block and ant exclusion by mycorrhizal treatment by block interactions as random factors to test for main effects using the appropriate error terms based on the above design (Littell et al. 2006). Data were log-transformed to achieve normality of residuals.

### Greenhouse test of VOC effects on ant attraction

#### Experimental design and measurements

To assess the effects of specific plant VOCs on ant attraction, we performed a dual-choice olfactometer experiment using workers of *Lasius niger* (Formicidae: Lasius) ants, the most common species at our study site as well as during the field experiment (X. Moreira, personal observation). We purchased the ant colony from Anthillshop (https://anthillshop.es/es/; Puerto de Santa María, Spain). The olfactometer is a two-sided system of glass tubes consisting of two vessels attached, on the opposite directions, to a central chamber by a 10-cm-long glass tube each (see Rasmann et al. 2014). Based on findings from the greenhouse study measuring AMF effects on plant VOCs, we prepared artificial emitters with one of two contrasting blends of synthetic VOCs using four chemical compounds (2-hexanol: Sigma-Aldrich, CAS number: 626–93-7; 3-carene: Sigma-Aldrich, CAS number: 13466–78-9; β-caryophyllene: Sigma-Aldrich, CAS number: 87–44-5; octanoic acid, octyl ester: Sigma-Aldrich, CAS number: 112–14-1). These compounds either exhibited significantly greater concentrations in AMF relative to non-AMF plants (e.g., 2-hexanol, 3-carene, and β-caryophyllene) or were abundant compounds regardless of the AMF treatment (> 10% of the total emission, e.g., octanoic acid, octyl ester) (see Table S1). We previously performed trials with different VOC mixtures to mimic the mean amounts (in ng h^−1^) of compounds emitted by potato plants in control and AMF-inoculated plants (see Results). We ended up using a “control” blend composed of 80 µL mL of 2-hexanol, 12 µL of 3-carene, 40 µL of β-caryophyllene, and 400 µL of octanoic acid, octyl ester in 10 mL of dichloromethane (Sigma-Aldrich, CAS number: 75–09-2), whereas the “mycorrhization” blend was composed of 100 µL of 2-hexanol, 20 µL of 3-carene, 120 µL of β-caryophyllene, 800 µL of octanoic acid, and octyl ester in 10 mL of dichloromethane. Artificial emitters consisted of 2 mL glass chromatographic vials topped by screw thread caps with a Teflon septum, which had a small hole cut in the center to pass a 12.5 cm capillary tube (100 μL ringcaps) (Moreira et al. 2018b). Each vial contained a small piece of cotton inoculated with 100 µL of control blend or 100 µL of mycorrhization blend. We placed artificial emitters with the control and mycorrhization blends on both sides of the olfactometer. Then, we placed one ant in the central chamber of the olfactometer, and recorded its first choice during a 5-min observation period (recorded as “1” for reaching a blend type of each replicate vs. “0” for not reaching it). We conducted the choice experiment 20 times and, in each case, used a different ant specimen. The VOC treatments were randomly alternated between left and right sides of the olfactometer to prevent influences of light and other unmeasured variables. In addition, the olfactometer was washed with ethanol and dried between trails to avoid potential effects of ant trails in the tubes. Furthermore, we previously conducted a control-choice test with vials lacking VOC blends and found that ants did not significantly prefer one side of the olfactometer over the other.

It is important to note that while exposing ants to artificial VOC blends is less realistic than using plants and/or real emissions, this approach allows for a precise control over the composition and concentration of VOCs. In this way, we were able to ensure the consistency in treatment application and reduce variability that might arise from differences in VOC emission between individual plants unrelated to mycorrhization, i.e., avoiding background noise in other volatile emissions due to other sources of variation. That said, we were only able to test for effects of two types of blends which limits inference that can be drawn given potential variation in AMF-induced VOCs (e.g., across variety by mycorrhizal treatment combinations).

### Statistical analyses

We analyzed the effect of VOC blend type (two levels: control vs. mycorrhization, fixed factor) on the odds of an ant’s first choice using a generalized linear mixed model with a binomial distribution and logit-link function (PROC GLIMMIX in SAS 9.4). Odds ratio values are the ratio between successful and unsuccessful events (i.e., ants choosing vs. not choosing a given blend type, respectively) for each treatment level, i.e., a likelihood of an ant being attracted to the control or mycorrhization volatile blend treatments.

## Results

### Greenhouse test of mycorrhizal treatment effects on plant traits

**Fig. 2:**
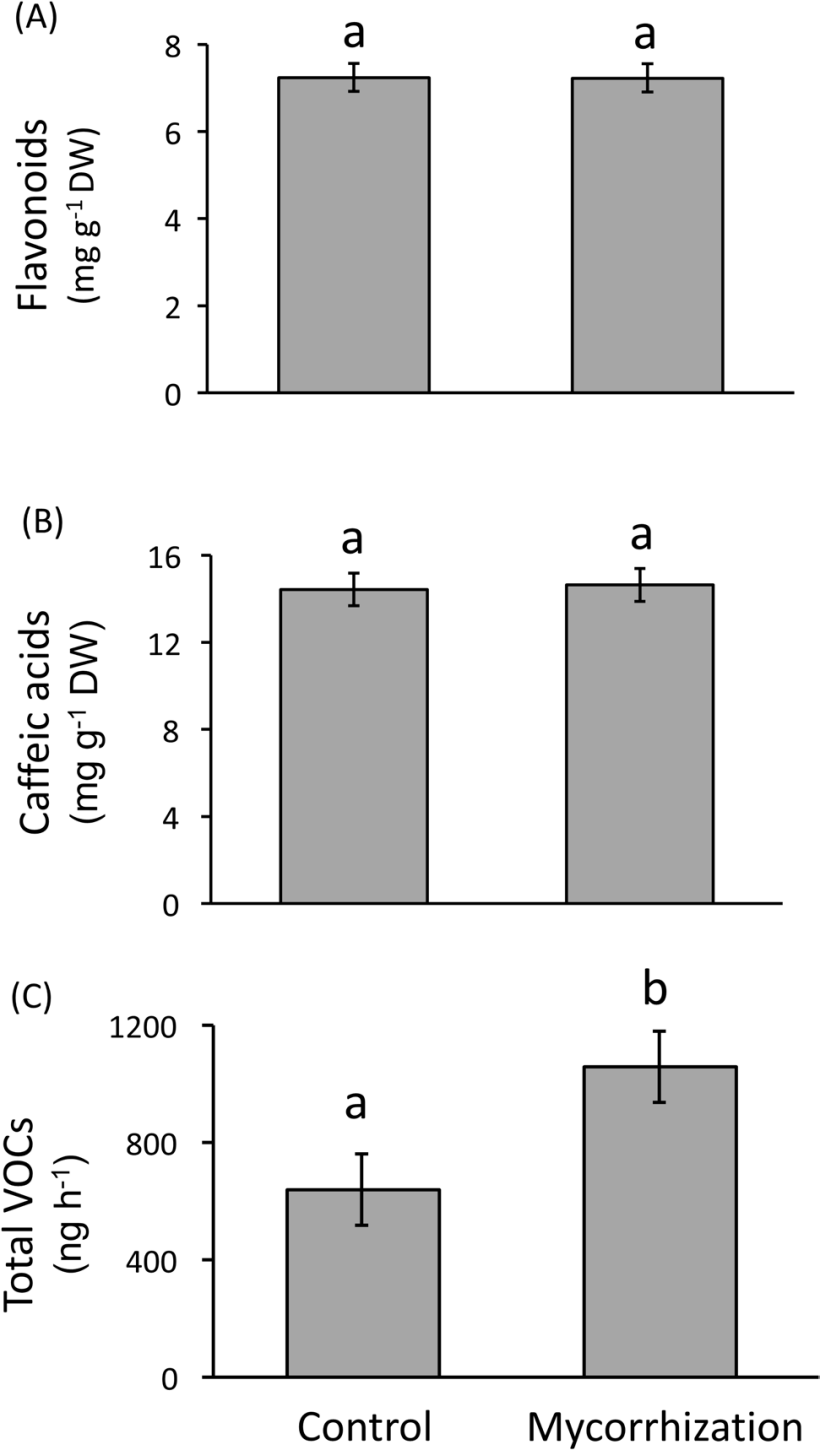
Effects of mycorrhizal treatment (two levels: control and mycorrhization) on the concentration (in mg g^−1^ DW) of leaf flavonoids (**A**) and caffeic acids (**B**), and total amount (in nanograms h^−1^) of VOCs (**C**) emitted by potato (*Solanum tuberosum*) plants. Bars are least square means ± SE obtained from linear models (*n* = 72 for phenolics and *n* = 24 for VOCs). Different letters above the bars indicate significant differences (*P* < 0.05) among mycorrhizal treatments. Statistics are shown in Table 1

We found that the percentage of root length colonized by AMF was 35.32 ± 2.46% for AMF-inoculated plants, whereas no detectable colonization was observed for control plants. Mycorrhization (vs. controls) significantly increased plant height (F_1,140_ = 6.97, *P* = 0.009), but not the number of leaves (F_1,140_ = 0.01, *P* = 0.941) (Fig. S2). In addition, we found no significant effect of mycorrhizal treatment on the concentration of leaf flavonoids (mean ± SE; control: 7.24 ± 0.32 mg g^−1^ DW, AMF-inoculated: 7.23 ± 0.32 mg g^−1^ DW) or caffeic acids (control: 14.42 ± 0.75 mg g^−1^ DW, AMF-inoculated: 14.63 ± 0.75 mg g^−1^ DW) (Table 1; Fig. 2A, B). In contrast, we found a significant effect of mycorrhizal treatment on plant total VOC emissions (Table 1), where AMF-inoculated plants emitted, on average, 1.7 times more total VOCs (1058.57 ± 121.43 ng h^−1^) than control plants (639.65 ± 121.43 ng h^−1^) (Fig. 2C). Although the PERMANOVA indicated no effect of mycorrhizal treatment on VOC composition (Table 1; Fig. S3), follow-up analyses on individual compounds showed that mycorrhization (vs. controls) significantly increased the emission of specific compounds over other, namely: 2-hexanol, 3-carene, and β-caryophyllene (Table S1), thus presumably altering blends to some extent. Analyses also indicated that plant varieties significantly varied with respect to plant height, the concentration of leaf caffeic acids, and VOC composition (Table 1; Fig. S4). Finally, there was a significant interaction between mycorrhizal treatment and plant variety on the concentration of caffeic acids (Table 1; Fig. S5).

**Table 1:** Results from linear models testing the effects of mycorrhizal treatment (two levels: control and mycorrhization), plant variety (three levels: Agria, Baraka, Desiree), and their interaction (all fixed factors) on plant growth-related variables (height, number of leaves), the concentration of leaf phenolic compounds (flavonoids and caffeic acids), and the total amount and composition of volatile organic compounds (VOCs) emitted by potato (*Solanum tuberosum*) plants

Response	Mycorrhizal treatment (M)			Plant variety (V)			M × V		
	DF	F/pseudo-F	*P*	DF	F/pseudo-F	*P*	DF	F/pseudo-F	*P*
Plant height	1, 138	7.10	**0.009**	2, 138	62.53	** < 0.001**	2, 138	2.35	0.099
Number of leaves	1, 138	0.01	0.941	2, 138	1.01	0.368	2, 138	0.30	0.744
Flavonoids	1, 136	0.00	0.976	2, 136	3.06	0.050	2, 136	0.99	0.375
Caffeic acids	1, 136	0.13	0.716	2, 136	6.06	**0.003**	2, 136	4.91	**0.009**
VOC emission	1, 42	5.95	**0.019**	2, 42	1.59	0.217	2, 42	0.65	0.529
VOC composition	1, 41	1.82	0.096	2, 41	2.46	**0.012**	2, 41	0.61	0.837

For VOC composition, we used a permutational multivariate analysis of variance (PERMANOVA) model. Plant sample size was 144 for growth variables and phenolics, and 48 for VOCs. Degrees of freedom (numerator, denominator), F-values (or Pseudo-F in the case of VOC composition) and associated significance levels (*P*) are shown. Significant *P* values (*P* < 0.05) are in boldface

### Field test of mycorrhizal and ant exclusion treatment effects on ant abundance and herbivory

**Fig. 3:**
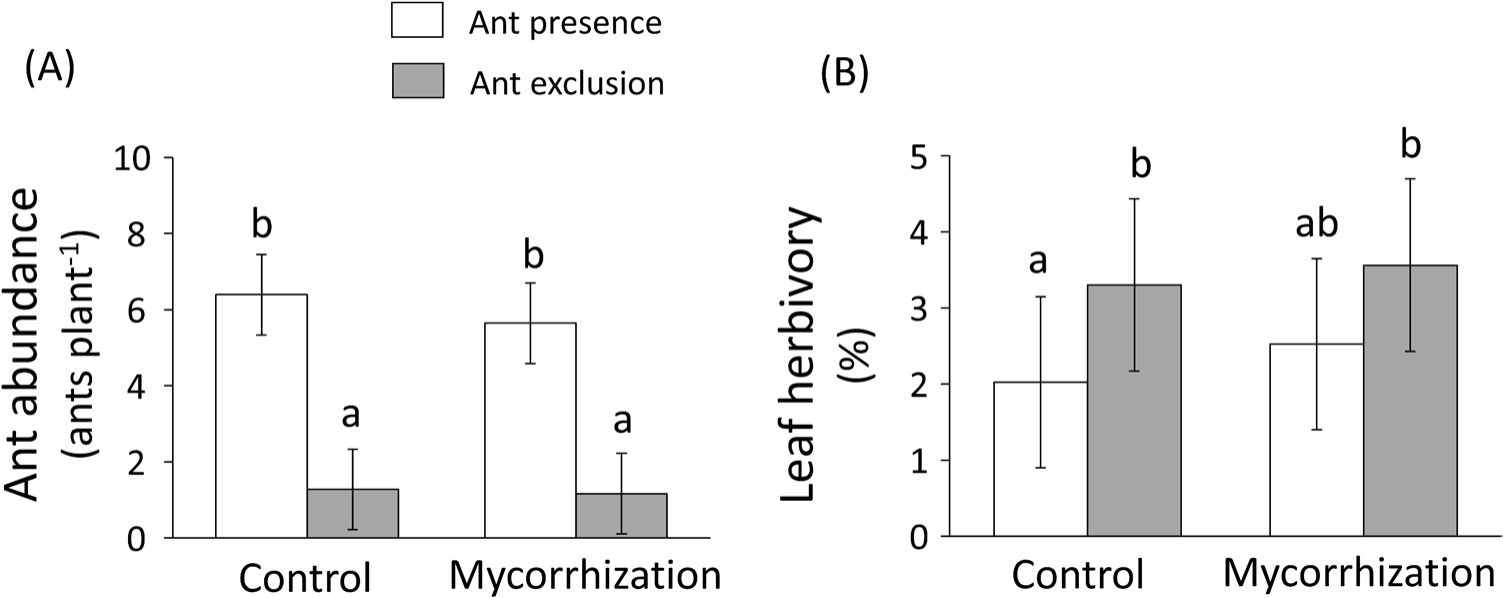
Effects of mycorrhizal treatment (two levels: control and mycorrhization) on **A** ant abundance (ants plant.^−1^) and **B** the percentage of leaf area consumed by chewing insects on potato (*Solanum tuberosum*) plants with ant presence (white bars) or subjected to an ant exclusion treatment (grey bars). Bars are back-transformations of log-transformed least-square means ± SE obtained from linear mixed models (*n* = 36). Different letters above the bars indicate significant differences (*P* < 0.05) among mycorrhizal and ant exclusion treatments. Statistics are shown in Table 2

The ant exclusion treatment significantly reduced ant abundance (back-transformed mean ± SE; ant-excluded: 1.22 ± 1.04 ants plant^−1^, non-excluded: 6.01 ± 1.04 ants plant^−1^) (Table 2, Fig. 3A), and this effect was consistent across mycorrhizal treatments (i.e., no significant interaction between ant exclusion and mycorrhizal treatments on ant abundance; Table 2, Fig. 3A). In addition, we found no significant effect of the mycorrhizal treatment on ant abundance (control: 2.85 ± 1.04 ants plant^−1^, AMF-inoculated: 2.56 ± 1.04 ants plant^−1^) (Table 2, Fig. 3A). The effect of plant variety was not significant in any case, but it did interact with the mycorrhizal treatment whereby the latter (negatively) influenced ant abundance in one variety but not in the others (Table 2; Fig. S5).

**Table 2:** Results from linear mixed models testing the effects of mycorrhizal treatment (two levels: control and mycorrhization), ant exclusion treatment (two levels: exclusion and presence), plant variety (three levels: Agria, Baraka, Desiree) and their two- and three-way interactions (all fixed factors) on ant abundance and percent leaf herbivory by insect chewers in potato (*Solanum tuberosum*) plants. We also included block as a random factor in the models

Factor	Ant abundance			Herbivory		
	DF	F	*P*	DF	F	*P*
Mycorrhizal treatment (M)	1, 6	3.58	0.107	1, 6	1.50	0.267
Ant exclusion treatment (A)	1, 3	789.27	** < 0.001**	1, 3	11.77	**0.041**
M × A	1, 6	0.08	0.783	1, 6	0.31	0.599
Plant variety (V)	2, 120	0.36	0.700	2, 109	0.30	0.741
M × V	2, 120	3.56	**0.031**	2, 109	0.01	0.994
A × V	2, 120	0.09	0.918	2, 109	1.90	0.155
M × A × V	2, 120	0.73	0.482	2, 109	1.50	0.267

We additionally included the ant exclusion by block and ant exclusion by mycorrhizal treatment by block interactions as random factors in order to analyze the main factors ant exclusion and mycorrhizal treatments with the appropriate error terms. We log-transformed herbivory data to achieve normality of the residuals. The number of plants was 144. Degrees of freedom (numerator, denominator), F-values, and associated significance levels (*P*) are shown. Significant *P* values (*P* < 0.05) are in boldface

We found no significant effect of the mycorrhizal treatment on the percent leaf area consumed by chewing insects (back-transformed mean ± SE; control: 2.60 ± 1.13%; AMF-inoculated: 3.00 ± 1.13%) (Table 2, Fig. 3B). In contrast, ant exclusion had a significant effect (Table 2), with ant-excluded plants exhibiting, on average, 52% higher herbivory (back-transformed mean ± SE: 3.43 ± 1.09%) compared to non-excluded plants (2.26 ± 1.09%) (Fig. 3B). However, we found no significant interaction between the mycorrhizal and ant exclusion treatments on herbivory (Table 2; Fig. 3B). Finally, there was no significant effect of plant variety as well as no significant (two- or three-way) interaction between this factor and the main effects (Table 2).

### Greenhouse test of VOC effects on ant attraction

**Fig. 4:**
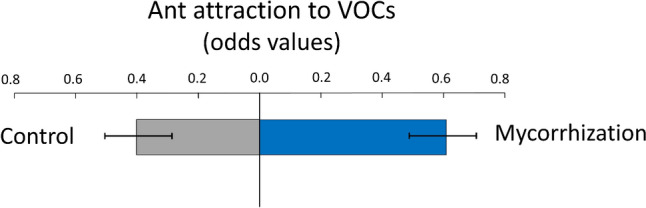
Ant (*Lasius niger*) attraction (measured as odds values) to synthetic blends of VOCs which mimicked the emission rates in response to mycorrhization (blue bars) or control (grey bars) emissions based on dual-choice assay using an olfactometer (see Methods). Bars are the mean odds ratio value (± SE) for each VOC exposure treatment obtained from a generalized linear mixed model (*n* = 20 assay replicates, see methods of experiment 2). Odds values were calculated as the ratio between successful and unsuccessful events (i.e., likelihood of an ant being attracted vs. not attracted to a blend type) for each replicate of each treatment level

Following from the mycorrhizal treatment effect on VOC emissions, and despite not having found an effect of AMF on ant abundance in the field, we conducted the dual-choice experiment with the olfactometer to test for effects of specific AMF-associated plant VOCs blends on *L. niger* attraction which could have gone undetected in the field. Nonetheless, consistent with the lack of AMF effects on ants in the field experiment, we found no significant difference in ant attraction (measured by the number of times chosen) between VOC blends mimicking emissions from AMF-inoculated plants and emissions from control plants (χ^2^ = 1.58, *P* = 0.209, df = 1; Fig. 4).

## Discussion

Despite having overall low levels of insect herbivory, we found evidence that ants reduced leaf damage on potato plants (as given a significantly lower proportion of leaf area removed on non-excluded vs. ant-excluded plants), whereas mycorrhization had no detectable influence on herbivory. Furthermore, and consistent with the latter finding, mycorrhization had no overall effect on plant direct defenses, and there was no evidence for mutualistic interactive effects on herbivory; i.e., AMF effects on herbivory were not mediated by ants. Consistently, while AMF drove an increase in VOC emissions, mycorrhization had no overall influence on ant abundance in the field or ant attraction to artificial VOC blends in the greenhouse. We therefore conclude that plant trait-mediated linkages between AMF and aboveground tri-trophic interactions were not present during our study.

Previous studies have predominantly reported positive effects of AMF on phenolic compounds, such as flavonoids and tannins (Dos Santos et al. 2017; Tekaya et al. 2022). While our findings overall disagree with these studies, it is important to note that we found evidence in one case (caffeic acids) that mycorrhization effects were contingent on the potato variety, with significant (and opposite) changes in two cases. Plant varieties differ in physiological or biochemical features impacting defense-related metabolic pathways (Agho et al. 2023), and responses appear to be compound type-specific, thus warranting further work to gain a more nuanced understanding of plant genetically based variation in AMF effects on direct chemical defenses in potato. Nonetheless, consistent with past studies showing that AMF drive increases in aboveground plant VOCs (van Dam et al. 2003; reviewed by Rasmann et al. 2017), we found that mycorrhization significantly elevated total potato VOC emissions, in this case consistently across potato varieties. This included increases in three individual VOCs of known ecological importance (2-hexanol, 3-carene and β-caryophyllene; Hare 2011). As examples, Copolovici et al. (2017) reported that mycorrhizal colonization enhanced VOC emissions in common myrtle (*Myrtus communis*), and Yuan et al. (2021) similarly demonstrated that AMF increased the emissions of VOCs in tomato (*Solanum lycopersicum*) plants, though in the latter case effects were contingent on the AMF species. Additional work measuring effects on other types of direct chemical defenses as well as nutrients, combined with the use of a larger number of plant varieties is needed to reach stronger conclusions. It is also important to bear in mind that our study was constrained to a single season and under relatively uniform abiotic conditions. Mycorrhization effects on plant traits have been shown to be temporally variable and highly context-dependent (Frew 2021; Qu et al. 2021), thus calling for future work exogenous factor manipulations (e.g., related to abiotic stress; Qu et al. 2021) that expand across seasons and in multiple sites.

Ants significantly influenced herbivory, with non-excluded plants showing a 34% lower mean value of leaf damage than excluded plants. The most abundant ant species was *L. niger*, a generalist species previously shown to provide protection against insect herbivores in other plant species such as *Senecio jacobaea* (Vrieling et al. 1991) and *Tanacetum vulgare* (Mehrparvar et al. 2018). However, counter to expectations, ant abundance was not affected by mycorrhization, and there was no evidence for mutualist interactive effects on herbivory. These results are consistent with the lack of effect of AMF-induced volatile blends on ant attraction. In contrast to these results, a study by Keller et al. (2018) found non-additive mutualist effects on the extrafloral nectar-bearing legume *C. fasciculata*. Specifically, rhizobia had a positive effect on arthropod abundance (possibly due to higher plant quality), but only when ants were excluded (Keller et al. 2018). When ants were present, they deterred or consumed insect prey, thereby neutralizing the bottom-up effects of rhizobia on the arthropod community (Keller et al. 2018). However, their study did not evaluate whether this shift in the arthropod community resulted in changes in herbivory. More broadly, it is important to emphasize that our study is the first to test soil microbe plant VOC-mediated effects on ants in a system lacking plant rewards (e.g., extrafloral nectar). To the extent that plant VOCs play a role in generalist ant–plant systems (as potato), addressing this knowledge gap could provide new insights into linkages between below- and aboveground plant-associated communities.

It is also worth noting that the mycorrhization effect on ant abundance varied significantly depending on the plant variety, resulting in a lower ant abundance in one case (Desiree) but no effect on the others. It is unclear how this effect arose and is presumably unrelated to differences in VOC responses to mycorrhization, which were consistent across varieties. Instead, it could be explained by varietal-dependent changes in unmeasured traits (e.g., trichomes, which interfere with ant foraging). Regardless, this particular case depicted a negative effect of AMF on ant abundance (rather than a positive one), which contradicts our initial hypothesis and ultimately did not trascend to parallel varietal contingency in mutualist individual and interactive effects on herbivory (i.e., there were non-significant interactions between variety and main effects on leaf damage). That said, we still think plant genetically based variation in trait responses to AMF and resulting interaction outcomes deserve further attention in this system, including tests with varietal-specific AMF-induced blends.

In studying plant VOC-mediated microbial effects on higher trophic levels, an important consideration is whether plants also offer rewards for parasitoids and predators, as this could influence ecological and evolutionary mechanisms and outcomes. Plant volatile effects on ants have been investigated in systems where plant rewards are found in either reproductive (e.g., flowers) or non-reproductive tissues (reviewed by Heil 2008). It has been pointed out that associative learning by ants in response to VOCs may be particularly strong when emissions correlate with the production of rewards such as extrafloral nectar (Nelson et al. 2019). That said, there are a few studies showing plant VOC effects on ants in systems where plants lack rewards. For instance, Rasmann et al. (2014) demonstrated that *Vicia sepium* plants at low elevations emitted more VOCs were visited by more ants (*Formica fusca, Formica selysi, Myrmica* spp.) and were less susceptible to herbivore attack. Similarly, Brouat et al. (2000) also found that VOCs emitted by *Leonardoxa africana* attracted *Petalomyrmex phylax* ants which in turn reduced insect herbivory. These VOC-mediated ant–plant interactions could be highly common, particularly in generalist systems, thus warranting further investigation. Besides reaching greater inference across systems, doing so can also feed from as well as enrich (via empirical tests) past discussions and theoretical frameworks on information- and reward-based mutualisms (Bronstein 1994, 2015), while contributing in broader terms to better understand plant-mediated microbe effects on the third trophic level and its consequences for top-down control.

On a closing note, we point out that while our study focused on AMF effects on ants, it is possible that ants also influence AMF or other soil microbes, leading to reciprocal effects. For example, Keller et al. (2018) described such reciprocal effects whereby rhizobia increased ant abundance via higher production of extrafloral nectaries, but ants in turn reduced rhizobia abundance (unknown mechanism) and also appeared to have contributed to fungal spore dispersal. These dynamics might be common and of central importance to understanding mutualist coexistence and their joint effects on plants and community structure. Longer-term studies replicated across contrasting sites are needed (particularly in long-lived plants) to incorporate spatiotemporal variation in biotic (e.g., ant and microbe species composition) and abiotic factors.

### Supplementary Information

Below is the link to the electronic supplementary material.

Supplementary file1 (DOCX 350 kb)

## Data Availability

The data used in this study will be archived at dryad repository upon publication.

## Abbreviations

AMF: Arbuscular mycorrhizal fungi
VOC: Volatile organic compound
