# Supplementary material for: Testing the joint effects of arbuscular mycorrhizal fungi and ants on insect herbivory on potato plants
Source: Planta. 2024 Jul 30;260(3):66. doi: 10.1007/s00425-024-04492-1 (PMC11289011; doi:10.1007/s00425-024-04492-1)
Supplement: Supplementary file 1 — Supplementary file1 (DOCX 350 kb) [file 425_2024_4492_MOESM1_ESM.docx]

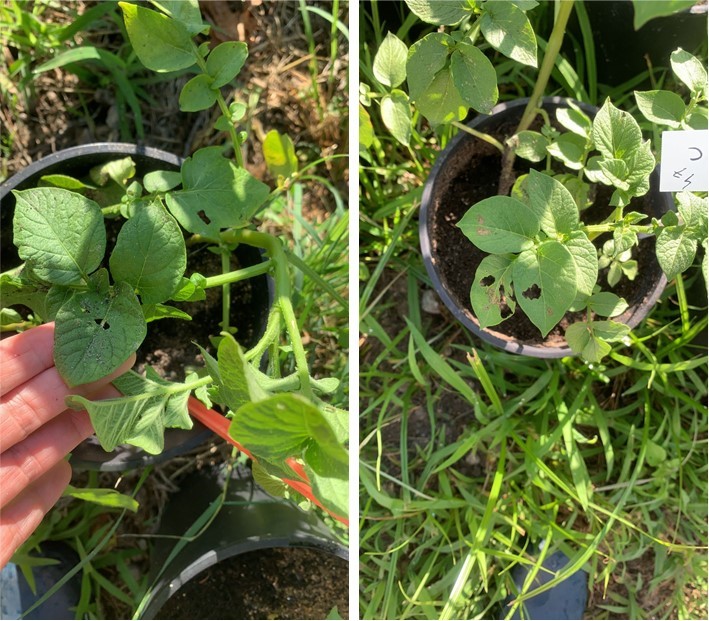


**Fig. S1** Examples of damage caused by insect leaf-chewing herbivores in our experimental potato (*Solanum tuberosum*) plants. Photo credits: Lucía Martín-Cacheda.

**Fig. S2** Effects of mycorrhizal treatment (two levels: control and mycorrhization) on **(A)** plant height (in cm) and **(B)** number of leaves in potato (*Solanum tuberosum*) plants. Bars are least square means ± SE (*n* = 72). Mycorrhizal treatment significantly increased plant height (F_1,138_ = 7.10, *P* = 0.009), but not the number of leaves (F_1,138_ = 0.01, *P* = 0.941). Different letters above the bars indicate significant differences between mycorrhizal treatments at *P* < 0.05.


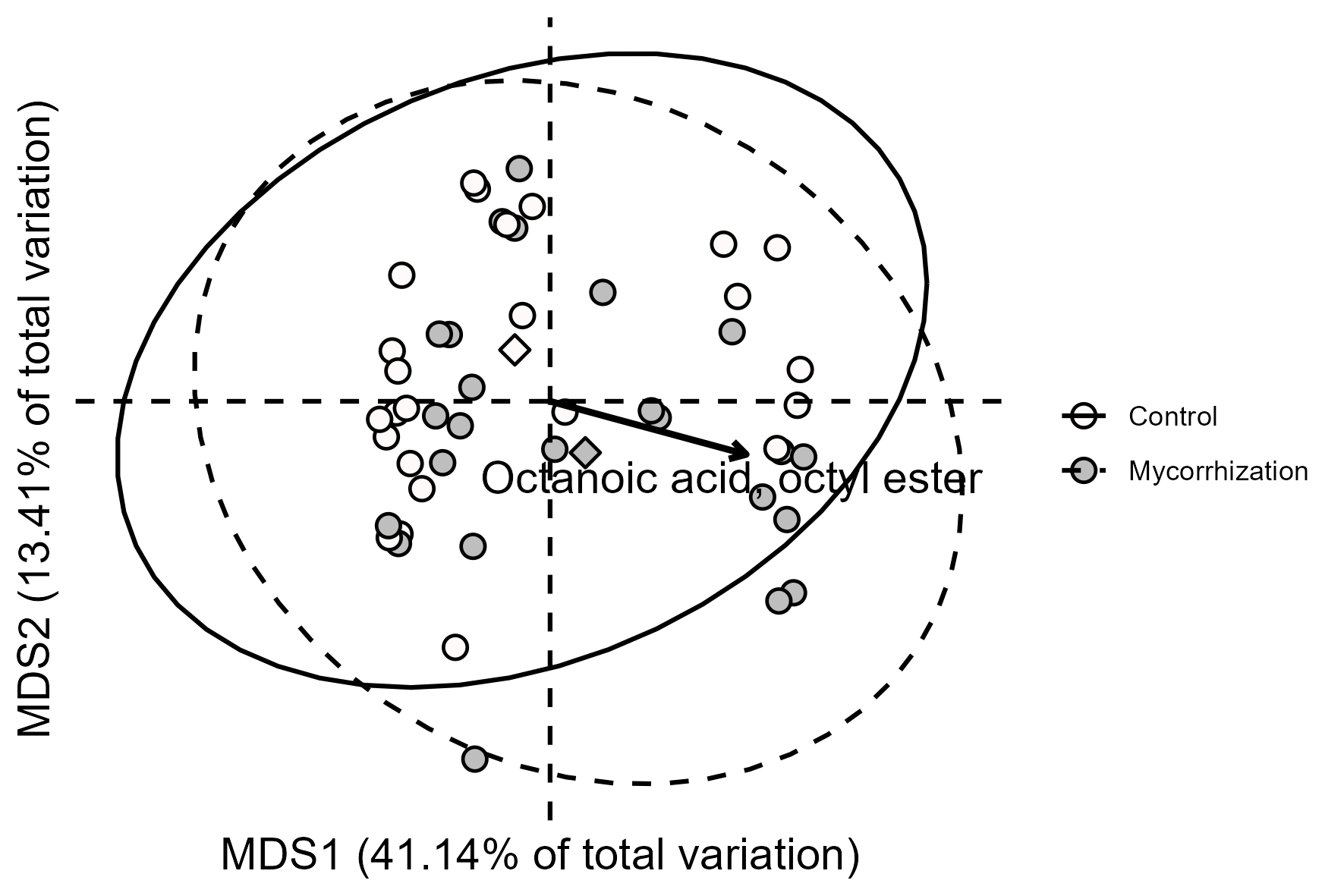


**Fig. S3** Unconstrained ordinations showing the effects of mycorrhizal treatment (two levels: control and mycorrhization) on the composition of volatile organic compounds produced by potato plants. Each point represents an individual plant (*n* = 48). Biplot arrows show associated linear trends with volatiles, scaled to reflect relative magnitude of effects based on R^2^ values (R^2^ > 0.50, *P* < 0.001). The mycorrhizal treatment ordination displays control and mycorrhization centroids (represented as diamonds) and 95% ellipses. The first two axes of this ordination accounted for 54% of the treatment effect in volatile composition (41% and 13%, respectively).

**Fig. S4** Effects of potato (*Solanum tuberosum*) variety (three levels: Agria, Baraka, Desiree) on **(A)** plant height (in cm) and **(B)** leaf concentration of caffeic acids (in mg g^-1^ DW). Bars are least square means ± SE (*n* = 48). Different letters above the bars indicate significant differences between potato varieties at *P* < 0.05.

**Fig. S5** Effects of mycorrhizal treatment (two levels: control and mycorrhization) on **(A)** leaf concentration of caffeic acids (in mg g^-1^ DW) and **(B)** ant abundance (ants plant^-1^) in potato (*Solanum tuberosum*) plants from three varieties (three levels: Agria, Baraka, Desiree). Bars are least square means ± SE in panel A and back-transformations of log-transformed least-square means ± SE in panel B (*n* = 24). Asterisks above the bars indicate significant differences among mycorrhizal treatments across plant varieties (*) *P* < 0.05, (**) *P* < 0.01

**Table S1** Means (± SE) for emission of individual volatile organic compounds (naphtalene-equivalent ng h^-1^) identified by GC-MS under two mycorrhizal treatments (control vs. mycorrhization by *Rhizoglomus irregulare*) in *Solanum tuberosum* plants^1^. We used 24 plants for each mycorrhizal treatment (48 plants in total). RT = Retention times. KRI = Kovats retention index used for identification of compounds without commercial standards (KRI_c_ for calculated values and KRI_e_ for expected values from the NIST database). F-values with the degrees of freedom (numerator, denominator), and associated significance levels (*P*) are shown. Significant *P*-values (*P* < 0.05) are in boldface.

| Compound | RT | KRIe | KRIc | Control | Mycorrhization | F_1,46_ | *P*-value |
| --- | --- | --- | --- | --- | --- | --- | --- |
| **2-hexanol** | 5.87 | 801 | 804.7 | **48.76 ± 2.18** | **59.86 ± 2.46** | 11.19 | **< 0.01** |
| α-pinene† | 9.46 | 937 | 934.9 | 21.77 ± 2.55 | 20.17 ± 2.28 | 0.04 | 0.851 |
| β-pinene | 10.59 | 979 | 975 | 6.48 ± 0.877 | 4.48 ± 0.67 | 1.07 | 0.307 |
| β-myrcene† | 11.04 | 991 | 990.9 | 13.71 ± 02.18 | 18.79 ± 2.55 | 2.06 | 0.158 |
| **3-carene†** | 11.53 | 1011 | 1008.7 | **7.11 ± 1.18** | **11.61 ± 0.96** | 15.34 | **< 0.001** |
| Eucalyptol† | 12.15 | 1032 | 1031.7 | 11.96 ± 1.27 | 12.08 ± 01.07 | 0.05 | 0.822 |
| 4-methyldecane | 12.84 | 1060 | 1057.3 | 15.11 ± 1.75 | 15.29 ± 1.5 | 0.02 | 0.894 |
| Linalool† | 14.04 | 1099 | 1102 | 7.01 ± 1.58 | 6.76 ± 1.22 | 0.09 | 0.760 |
| Nonanal† | 14.14 | 1104 | 1105.9 | 20.76 ± 5.15 | 20.36 ± 3.53 | 0.38 | 0.540 |
| 1,3,7-nonatriene, 4,8-dimethyl-, (3E)- | 14.43 | 1116 | 1117.4 | 10.69 ± 4.95 | 8.88 ± 2.69 | 0.06 | 0.805 |
| (E)-2-Nonenal | 15.61 | 1162 | 1165.8 | 4.22 ± 0.60 | 4.64 ± 0.84 | 0.00 | 0.968 |
| Dodecane† | 16.49 | 1200 | 1199.1 | 14.96 ± 1.86 | 14.78 ± 1.32 | 0.19 | 0.666 |
| Decanal | 16.84 | 1210 | 1213.9 | 7.41 ± 1.11 | 6.92 ± 0.69 | 0.12 | 0.729 |
| Tridecane† | 18.86 | 1300 | 1299.5 | 12.02 ± 2.08 | 12.40 ± 1.31 | 1.02 | 0.317 |
| α-copaene† | 20.56 | 1376 | 1376.2 | 4.83 ± 0.49 | 5.79 ± 0.38 | 3.46 | 0.069 |
| Tetradecane† | 21.06 | 1400 | 1398.7 | 7.87 ± 0.91 | 8.94 ± 0.76 | 1.65 | 0.205 |
| α-gurjunene | 21.29 | 1409 | 1409.7 | 5.63 ± 0.94 | 7.35 ± 1.31 | 1.83 | 0.182 |
| **β-caryophyllene†** | 21.52 | 1419 | 1420.7 | **22.51 ± 4.64** | **66.03 ± 14.86** | 13.90 | **< 0.001** |
| α-bergamotene | 21.88 | 1435 | 1437.9 | 3.73 ± 0.99 | 4.91 ± 0.88 | 1.26 | 0.268 |
| cis-β-farnesene | 22.06 | 1444 | 1446.6 | 34.57 ± 13.27 | 39.38 ± 7.21 | 2.00 | 0.164 |
| 5,9-undecadien-2-one, 6,10-dimethyl- | 22.25 | 1456 | 1455.7 | 4.98 ± 0.85 | 5.95 ± 2.16 | 0.02 | 0.889 |
| (E)-β-famesene† | 22.31 | 1457 | 1458.5 | 3.90 ± 0.80 | 7.93 ± 1.89 | 3.52 | 0.067 |
| β-santalene | 22.46 | 1462 | 1467.5 | 28.92 ± 4.15 | 63.82 ± 20.02 | 0.01 | 0.929 |
| γ-gurjenene | 22.67 | 1473 | 1475.8 | 14.95 ± 4.06 | 12.65 ± 2.85 | 0.79 | 0.379 |
| Germacrene D | 22.85 | 1481 | 1484.4 | 14.95 ± 4.06 | 39.74 ± 14.63 | 3.21 | 0.080 |
| β-Selinene | 23 | 1486 | 1491.6 | 7.11 ± 2.31 | 11.12 ± 2.73 | 0.32 | 0.575 |
| Pentadecane | 23.07 | 1500 | 1494.9 | 14.64 ± 5.37 | 6.05 ± 1.54 | 0.01 | 0.938 |
| α-farnesene† | 23.41 | 1508 | 1511.8 | 3.54 ± 0.97 | 2.11 ± 0.77 | 0.81 | 0.373 |
| β-sesquiphellandrene | 23.71 | 1524 | 1527.1 | 4.88 ± 1.47 | 7.53 ± 1.42 | 3.41 | 0.071 |
| (-)-globulol | 24.79 | 1580 | 1581.9 | 7.19 ± 2.39 | 13.11 ± 7.03 | 0.00 | 0.974 |
| Viridiflorol | 24.97 | 1591 | 1591 | 8.25 ± 1.02 | 38.52 ± 21.41 | 2.44 | 0.125 |
| Widdrol | 25.36 | 1610 | 1611.4 | 7.42 ± 1.5 | 37.9 ± 27.82 | 2.37 | 0.130 |
| Octanoic acid, octyl ester | 28.41 | 1779 | 1778.3 | 226.28 ± 65.28 | 437.77 ± 6.16 | 3.23 | 0.079 |
| Hexadecanal | 29.08 | 1817 | 1816.8 | 5.79 ± 0.82 | 6.16 ± 1.09 | 0.15 | 0.704 |

^1^We performed *P*-value adjustments using the False Discovery Rate for *P* < 0.05 to avoid inflating Type I error due to multiple testing.

^†^Compounds identified with commercial pure standards.
